# West Texas Medical Association

**Published:** 1902-04

**Authors:** W. B. Russ

**Affiliations:** Secretary


### West Texas Medical Association.

The last regular meeting of the West Texas Medical Association was the best attended and most enthusiastic held by this society in many years.

A committee consisting of Drs. Paschal, Spring, Moss, Kingsley and Jackson, on behalf of the association, presented a handsomely engraved testimonial containing resolutions of respect and admiration and conferring an honorary life membership upon Dr. Ferdinand Herff. This testimonial was signed by the officers and by more than thirty of the members. In response Dr. Herff expressed his great pleasure at receipt of the honor. He had never before, he said, known the association, since its foundation in 1876, to be in so prosperous a condition as it is at the present time. He believes the organization now compares favorably with any similar body in this or any other State.

The program was one of great interest. Dr. Paschal presented for clinical study a case of locomotor ataxia, and then read a paper upon resection of the knee joint. Dr. A. S. McDaniel led the discussion. Drs. F. Herff, Wolf, Lankford, King, Burg and Jackson took part in it.

Dr. E. H. Elmendorf exhibited several pathological specimens, the most interesting being a specimen of cancer of the lesser curvature of the stomach with extensive secondary deposits in the liver.

At every meeting of the association during the past six months from one to five cases have been presented for clinical study. Cases for clinical study and pathological specimens are now included as numbers on the regular program.

W. B. Russ, M. D., Secretary.

